# Dataset of surface plasmon resonance based on photonic crystal fiber for chemical sensing applications

**DOI:** 10.1016/j.dib.2018.05.026

**Published:** 2018-05-18

**Authors:** Md. Abdul Khalek, Sujan Chakma, Bikash Kumar Paul, Kawsar Ahmed

**Affiliations:** a Department of Information and Communication Technology, Mawlana Bhashani Science and Technology University, Santosh, Tangail 1902, Bangladesh; b Group of Bio-photomatiχ, Bangladesh; c Department of Software Engineering, Daffodil International University, Sukrabad, Dhanmondi, Dhaka 1207, Bangladesh

## Abstract

In this research work a perfectly circular lattice Photonic Crystal Fiber (PCF) based surface Plasmon resonance (SPR) based sensor has been proposed. The investigation process has been successfully carried out using finite element method (FEM) based commercial available software package COMSOL Multiphysics version 4.2. The whole investigation module covers the wider optical spectrum ranging from 0.48 µm to 1.10 µm. Using the wavelength interrogation method the proposed model exposed maximum sensitivity of 9000 nm/RIU(Refractive Index Unit) and using the amplitude interrogation method it obtained maximum sensitivity of 318 RIU^−1^. Moreover the maximum sensor resolution of 1.11×10^−5^ in the sensing ranges between 1.34 and 1.37. Based on the suggested sensor model may provide great impact in biological area such as bio-imaging.

**Specification Table**

**Table:** 

Subject area	Sensor
More Specific Subject area	Surface Plasmon Resonance based biosensor.
Types of data	Numerical analysis
How data was acquired	Full vectorial finite element method (FV-FEM) based tool COMSOL Multiphysics version 4.2 with circular PML.
Data format	Raw data, tables, figures.
Data accessibility	Data within the article and GitHub. Link:

**Value of the data**

• A PCF based SPR sensors are highly used in clinical diagnosis and the biomedical engineering based devices. The SPR based sensor has been proposed to enhance the performance of the sensor system.
• Derived numerical can assist the engineers, researchers, scientist, those who are especially interested with SPR based chemical.
• The presented simple designs and data analysis can support the researchers to reduce the complexity and implement high robust SPR sensor designs.
• Dataset is highly suitable for the benchmark of different liquid as well as chemical sensing application using PCF based SPR sensor.
• Presented senor model is experienced with superior performance than the previous existing sensor model.

## 1 Data

This article demonstrates the implementation of the photonic crystal Fiber (PCF) based sensor with cross sectional view. Table 1 is illustrating the data set for gold thickness of the structure; Table 2 is demonstrating the dataset for PML depth on fiber properties; Table 3 is describing about different chemical area; Table 4 is illustrating the data set for various radius of the center air hole; Table 5 is describing the dataset for different pitch value.

**Table 1:** Variations on several Gold thicknesses to observe the modal properties of the proposed PCF the operating wavelength lambda (*λ*)=0.48 µm to 1.10 µm. The diameter of center air hole at the first layer and other two air hole at the second layer are *r*_c_=*r*_2_=0.2 µm. The rest air holes are denoted by *r*_1_ where, *r*_1_=0.4 µm. The air holes inside the ring are organized by maintaining a fixed distance (*p*) where, *p*=1.8 µm. The thickness *d*_g_ of the gold layer, analyte layer and PML layer is 30–40 nm, 0.965 µm and 7.2 µm respectively.

Gold thickness (nm)	Analyte (*n*_a_)	Peak Loss (dB/cm)	Amplitude sensitivity (RIU^−1^)
30	1.36	172.32 (at *λ*=0.51 µm)	240.0451
	1.37	397.06 (at *λ*=0.59 µm)	
35	1.36	375.49 (at *λ*=0.62 µm)	318.1160
	1.37	700.04 (at *λ*=0.71 µm)	
40	1.36	424.01 (at *λ*=0.72 µm)	288.9673
	1.37	537.49 (at *λ*=0.51 µm)

**Table 2:** Variations of several PML depth to observe the modal properties of the proposed PCF; the operating wavelength lambda (*λ*)=0.48 µm to 1.10 µm and gold thickness 35 nm. The diameter of center air hole at the first layer and other two air hole at the second layer are *r*_c_=*r*_2_=0.2 µm. The rest air holes are denoted by *r*_1_ where, *r*_1_=0.4 µm. The air holes inside the ring are organized by maintaining a fixed distance (*p*) where, *p*=1.8 µm. The thickness *d*_g_ of the gold layer, analyte layer and PML layer is 35 nm, 0.965 µm and 7.0–7.4 µm respectively.

PML depth (µm)	Wavelength (µm)	Analyte (*n*_a_)	Peak Loss (dB/cm)
7.0	0.56	1.35	106.17
	0.62	1.36	374.76
7.2	0.56	1.35	106.34
	0.62	1.36	375.49
7.4	0.72	1.35	374.81
	0.84	1.36	106.17

**Table 3:** Variations of several chemical area to observe the modal properties of the proposed PCF; the operating wavelength lambda (*λ*)=0.48 µm to 1.10 µm, gold thickness 35 nm and PML 7.2 µm.

Chemical area (µm)	Analyte	Peak Loss (dB/cm)	Amplitude sensitivity (RIU^−^^1^)
0.565	1.35	106.17 (at *λ*=0.56 µm)	269.8864
	1.36	374.76(at *λ*=0.62 µm)	
0.965	1.35	106.34(at *λ*=0.56 µm)	318.1160
	1.36	375.49(at *λ*=0.62 µm)	
1.365	1.35	106.17(at *λ*=0.56 µm)	291.3925
	1.36	374.81(at *λ*=0.62 µm)

**Table 4:** Variations of several radius of center air hole to observe the modal properties of the proposed PCF; the operating wavelength lambda (*λ*)=0.48 µm to 1.10 µm, gold thickness 35 nm, PML 7.2 µm and chemical area 0.965 µm.

Center air hole radius (µm)	Analyte (*n*_a_)	Peak Loss(dB/cm)	Amplitude sensitivity (RIU^−1^)
0.1	1.35	104.39 (at *λ*=0.56 µm)	290.1938
	1.36	367.89(at *λ*=0.62 µm)	
0.2	1.35	106.34 (at *λ*=0.56 µm)	318.1160
	1.36	375.49(at *λ*=0.62 µm)	
Without center	1.35	103.99 (at *λ*=0.56 µm)	289.9285
	1.36	366.34 (at *λ*=0.62 µm)

**Table 5:** Variations of several pitch value to observe the modal properties of the proposed PCF; the operating wavelength lambda (*λ*)=0.48 µm to 1.10 µm, gold thickness 35 nm, PML 7.2 µm, chemical area 0.965 µm and radius of center air hole 0.2 µm.

Pitch (µm)	Analyte (*n*_a_)	Peak Loss (dB/cm)	Amplitude sensitivity (RIU^−^^1^)
1.50	1.35	276.85 (at *λ*=0.55 µm)	231.6415
	1.36	807.90 (at *λ*=0.61 µm)	
2.00	1.35	106.34 (at *λ*=0.56 µm)	318.1160
	1.36	375.49 (at *λ*=0.62 µm)	
2.50	1.35	52.61 (at *λ*=0.57 µm)	261.3937
	1.36	175.49 (at *λ*=0.62 µm)	

The data which describes above tables are comparable with the articles [1], [2], [3].

## 2 Experimental design, materials and methods

**Fig. 1:**
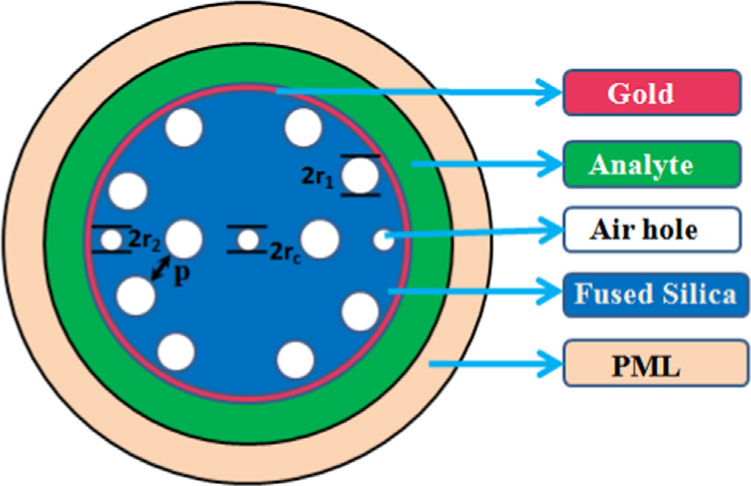
The cross sectional end faced view of the proposed PCF based SPR sensor.

Recently, various kinds of SPR based structures are also proposed [4], [5], [6] to obtain the high performance. Fig. 1(a) shows a circular lattice PCF sensor structure of cross sectional view. There have two layers of air holes in this structure where two air holes are missing in each layer. Comparatively two small air holes are placed in the second ring and one air hole is placed in the center. Here in the proposed structure, the distance between center-to-center is defined by the p, the radius of the center air hole is defined by *r*_c,_ *r*_2_ is defined as the radius of the small air holes which is equal to *r*_c_, *r*_1_ is the radius of rest of the air holes and the thickness of the gold layer is defined by *d*_g_. A larger central air-hole *r*_c_ is used to reduce the effective index of the core guided and as a result deteriorate the guidance along the core [7]. The gold film layer is placed at the outside of the fused silica layer where the thickness *d*_g_ of the gold film layer is 35 nm. The analyte layer is placed outside the gold film layer which thickness is 0.965 µm. In this raised structure the size of *r*_1_ is 0.4 µm. Last outer most layers are Perfectly Match Layer (PML) which thickness is 7.2 µm. The back ground layer of the structure is fused silica. Fig. 1(b) and (c) presents the surface mode and at wavelength *λ*=0.70 µm and *n*_a_ =1.37 nm.

In this raised structure we used a thin gold layer as an active plasmonic material outside the outermost air holes layer. Since gold is chemically inactive in hydrous atmosphere and represents rich resonance peak shift [8]. An analyte layer is also used outside the gold layer which will help to make a fiber structure easier and straight forward for fabrication process. we considered only one fiber core mode in the data set because this core mode is only eligible to provide high performance. On the other side, another mode provides abject performance and is not presence for all wavelength *λ* (lambda). That's why we neglect another mode. By following step by step analyzing process the operating selected mode can be achieved.

The following Sellmeier equation [8] is used to obtain the refractive index,

(1) $$n^{2}(\lambda )\;=\;1\;+\;\frac{B_{1}\lambda^{2}}{\lambda^{2}-\;C_{1}}\;+\;\frac{B_{2}\lambda^{2}}{\lambda^{2}-\;C_{2}}\;+\;\frac{B_{3}\lambda^{2}}{\lambda^{2}-\;C_{3}}$$

where *n* is denoted refractive index of fused silica that dependent on wavelength (*λ*), *λ* is the wavelength in µm. *B*_1_, *B*_2_, *B*_3_, *C*_1_, *C*_2_ and *C*_3_ are denoted the Sellmeier constants. The values of corresponding constants are respectively 0.69616300, 0.407942600, 0.897479400, 0.00467914826, 0.0135120631, and 97.9340025 for fused silica.

The following Drude–Lorenz model [9] is used to obtain the dielectric constant of the gold,

(2) $$\epsilon_{\mathrm{Au}}\;=\;\epsilon_{\propto }\;-\;\frac{\omega_{D}^{2}}{\omega (\omega \;+\;j\gamma D)}\;-\;\frac{∆\epsilon \;.\;\Omega_{L}^{2}}{\left(\omega^{2}\;-\;\Omega_{L}^{2}\right)\;+\;j\tau \omega }$$

where the permittivity of gold is denoted by *ϵ*_Au_, ϵα is the permittivity at high frequency that has a value of 5.9673, ω is the angular frequency that is defined as *ω*=2*πc*/*λ*, *c* is the velocity of light in vacuum, *ω*_D_ is denoted the plasma frequency, the damping frequency is denoted by *γD*, where *ω*_D_=4227.2*π* THz, *γD*=31.84*π* THz and weighting factor ∆*ϵ*=1.09. The spectral width *ΓL*=209.72*π* THz and oscillator strength *Ω*_L_=1300.14*π* THz respectively.

The following equation [10] is used to obtain the sensor's performance,

(3) α[dBm]=8.686×k0.Im[neff]×104

where *k*_0_=2*π*/*λ* is denoted the number of free space, operating wavelength is denoted by *λ* and the imaginary part of the effective refractive index denoted by Im(*n*_eff_).

To obtain the sensitivity of the PCF-based SPR sensor the following formula [11] is used,

(4) $$S_{\lambda }\left(\mathrm{nm}/\mathrm{RIU}\right)\;=\;∆\lambda_{\mathrm{peak}}/∆n_{a}$$

where ∆*λ*_peak_ is used to indicate the distinction of wavelength peak shifts and ∆*n*_a_ is used to indicate the difference of analyte refractive index RI.

To obtain the resolution of the raised structure the following formula [12] is used,

(5) $$R\left(\mathrm{RIU}\right)\;=\;∆n_{a}\;*\;∆\lambda_{\min}/∆\lambda_{\mathrm{peak}}$$

where ∆*n*_a_=0.01, ∆*λ*_min_=0.1 nm, and ∆*λ*_peak_=90 nm; as a result a high value of sensor resolution is obtained as high as 1.11×10^−5^.

The following formula [13] is used to obtain the amplitude sensitivity,

(6) $$S_{A}\left(\lambda \right)\left[{\mathrm{RIU}}^{-1}\right]\;=\;\;-\;\frac{1}{\alpha (\lambda ,n_{a})}\frac{\partial \alpha (\lambda ,n_{a})}{\partial n_{a}}$$

where *α*(*λ*, *n*_a_) is denoted the overall propagation loss at a specific refractive index (RI) of analyte and *∂α*(*λ*, *n*_a_) is indicated the difference between the two loss spectra.

**Fig. 2:**
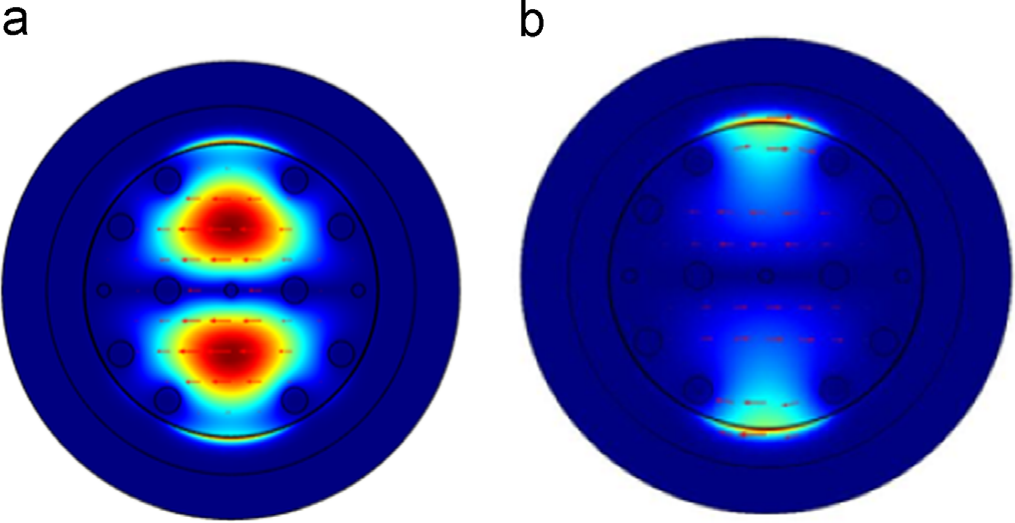
(a) Surface mode and (b) SPP mode of proposed structure for *λ*=0.70 µm and *n*_a_=1.37 nm.

**Fig. 3:**
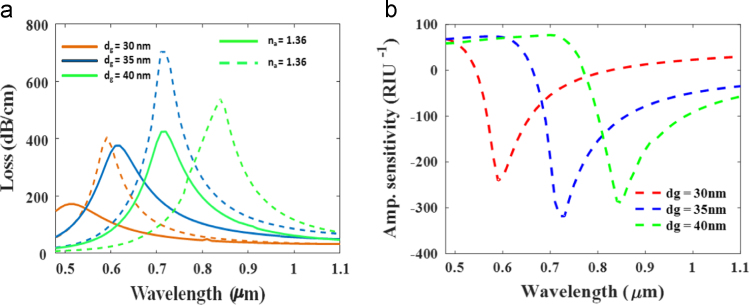
(a) Confinement loss variation for different thickness of gold layer and (b) amplitude sensitivity for different thickness of gold layer with *p*=2 µm, *r*_c_=*r*_2_=0.2 µm, *r*_1_=0.4 µm for analyte RI of 1.36.

Fig. 3(a) shows the consequent loss spectra for different gold layer thickness at analyte 1.36 and 1.37 as described in Table 1. From this analysis we can see that the proposed structure provide highest loss for gold thickness 35 nm. On the other hand, Fig. 2(b) presents the corresponding amplitude sensitivity with the variation of gold thickness. It easily clarify that the proposed structure is also provides highest amplitude sensitivity for gold layer thickness 35 nm.

## Financial support

No financial support was provided to any of the authors for this research work.

## References

[1] Liedberg B., Nylander C., Lunström I. (1983). Surface plasmon resonance for gas detection and biosensing. Sens. Actuators.

[2] Qin W., Li S., Yao Y., Xin X., Xue J. (2014). Analyte-filled core self-calibration microstructured optical fiber based plasmonic sensor for detecting high refractive index aqueous analyte. Opt. Lasers Eng..

[3] Dash J.N., Jha R. (2014). SPR biosensor based on polymer pcf coated with conducting metal oxide. IEEE Photon. Technol. Lett..

[4] Azzam S.I. (2016). Multichannel photonic crystal fiber surface plasmon resonance based sensor. Opt. Quantum Electron..

[5] Yang X. (2017). Analysis of graphene-based photonic crystal fiber sensor using birefringence and surface plasmon resonance. Plasmonics.

[6] Gangwar R.K., Singh V.K. (2016). Highly sensitive surface plasmon resonance based D-shaped photonic crystal fiber refractive index sensor. Plamonics.

[7] Dash J.N., Jha R. (2014). Graphene-based birefringent photonic crystal fiber sensor using surface plasmon resonance. IEEE Photonics Technol. Lett..

[8] Hasan M.R., Hasan M.I., Anower M.S. (2015). Tellurite glass defectcore spiral photonic crystal fiber with low loss and large negative flattened dispersion over S + C + L + U wavelength bands. Appl. Opt..

[9] Improved Analytical Fit of Gold Dispersion: Application to the Modeling of Extinction Spectra with a Finite-difference Time-domain Method. (Vial).

[10] Aoni R.A., Ahmed R., Alam M.M., Razzak S.A. (2013). Optimum design of a nearly zero ultra-flattened dispersion with lower confinement loss photonic crystal fibers for communication systems. Int. J. Sci. Eng. Res..

[11] Akowuah E.K., Gorman T., Ademgil H., Haxha S., Robinson G.K., Oliver J.V. (2012). Numerical analysis of a photonic crystal fiber for biosensing applications. IEEE J. Quantum Electron..

[12] Wang G., Li S., An G., Wang X., Zhao Y., Zhang W., Chen H. (2016). Highly sensitive D-shaped photonic crystal fiber biological sensors based on surface plasmon resonance. Opt. Quantum Electron..

[13] Lu Y. (2013). SPR sensor based on polymer photonic crystal fibers with metal nanolayers. Sensors.

