# Supplementary material for: Dataset of surface plasmon resonance based on photonic crystal fiber for chemical sensing applications
Source: Data Brief. 2018 May 18;19:76–81. doi: 10.1016/j.dib.2018.05.026 (PMC5993009; doi:10.1016/j.dib.2018.05.026)
Supplement: Supplementary file 1 — Supplementary material [file mmc1.docx]

**Conflicts of Interest Statement**

2018-04-25

**The Chief Editor**

**Data in Brief, Elsevier.**

**Dear Editor,**

On behalf of all the authors, Me Bikash Kumar Paul would like to ask you to consider our manuscript entitled “**Dataset of Surface Plasmon Resonance Based on Photonic Crystal Fiber for Chemical Sensing Applications**” for publication in your journal as research article. In this paper a PCF based SPR sensor has been proposed which can be used as a chemical sensor or acquous analytes sensor. Using the wavelength interrogation method the proposed model exposed maximum sensitivity of 9000nm/RIU(Refractive Index Unit) and using the amplitude interrogation method it obtained maximum sensitivity of 318 RIU^-1^. Moreover the maximum sensor resolution of 1.11×10^-5^ in the sensing ranges between 1.34-1.37. Based on the suggested sensor model may provide great impact in biological area such as bio-imaging.

This manuscript has not been published yet and not even under consideration for publication elsewhere. All the authors have read the manuscript and approved this for submission as well as no conflict interests.

Sincerely,

Bikash Kumar Paul

Member of IEEE,

Department of Information and Communication Technology

Mawlana Bhashani Science and Technology University

Santosh, Tangail-1902, Bangladesh

Cell: +88 01520102707

Email: bikash.k.paul@ieee.org; bikash.swe@diu.edu.bd; bikash12019@gmail.com
